# High Frequency of HHV-7 Reactivation in Neurological and Neuropsychiatric Long COVID: A Retrospective Observational Study

**DOI:** 10.3390/v18091043

**Published:** 2026-09-20

**Authors:** Diane Ducret, Tatjana Mijatovic

**Affiliations:** 1 Independent Researcher, 75012 Paris, France; 2 BelgiumR.E.D. Laboratories S.A., Z1 Researchpark 100, B-1731 Asse, Belgium

**Keywords:** HHV-7, long COVID, herpesvirus reactivation, neuroinflammation, ME/CFS, post-viral syndrome

## Abstract

The neurological and neuropsychiatric manifestations of long COVID remain incompletely understood. Reactivation of latent herpesviruses has been proposed as a contributing mechanism, but human herpesvirus 7 (HHV-7) has received limited attention despite its neurotropic potential. We conducted a retrospective analysis of HHV-7 polymerase chain reaction (PCR) results in 30 patients (18 women and 12 men; age range, 21–55 years) with a neurological/neuropsychiatric long COVID phenotype tested within 24 months of SARS-CoV-2 infection at R.E.D. Laboratories SA, Belgium. HHV-7 positivity was compared with a historical pre-COVID-19 laboratory reference cohort comprising 320 diagnostic tests performed from 2012 to 2019. HHV-7 positivity was 29.1% (93/320; 95% CI, 24.4–34.3%) in the historical reference cohort and 76.7% (23/30; 95% CI, 59.1–88.2%) in the neurological/neuropsychiatric long COVID cohort. The between-cohort difference was significant using a two-sided Fisher’s exact test (*p* < 0.000001). All six patients tested within the first 12 months were HHV-7-positive. HHV-7-positive patients frequently reported neuropsychiatric symptoms, and in one longitudinal index case, viral load tracked self-reported symptom severity. In this patient-initiated retrospective study, HHV-7 positivity was substantially more frequent in the long COVID cohort than in the historical reference cohort. These findings are hypothesis-generating and support prospective investigation of HHV-7 in neurological and neuropsychiatric long COVID.

## 1. Introduction

Persistent neurological and neuropsychiatric symptoms following acute infection with severe acute respiratory syndrome coronavirus 2 (SARS-CoV-2), commonly referred to as long COVID, represent a major and still incompletely understood public health challenge [1,2]. A substantial proportion of patients report cognitive impairment (“brain fog”), fatigue, sleep disturbance, dysautonomia, and affective symptoms months to years after the initial infection [1,2,3]. Despite increasing recognition of these manifestations, their underlying biological mechanisms remain incompletely defined [1,2].

One hypothesis that has gained traction is that long COVID may, in part, reflect the reactivation of latent viruses under conditions of immune dysregulation [1,2]. During acute COVID-19, multiple studies have documented the reactivation of herpesviruses, including Epstein–Barr virus (EBV), cytomegalovirus (CMV), and human herpesvirus 6 (HHV-6), particularly in hospitalized or critically ill patients [4,5,6]. These reactivations are generally interpreted as a consequence of systemic inflammation, lymphocyte dysfunction, and immune exhaustion associated with severe viral infection.

In contrast, human herpesvirus 7 (HHV-7), a closely related betaherpesvirus within the Roseolovirus genus, has received comparatively little attention. HHV-7 is highly prevalent worldwide and establishes lifelong latency primarily in CD4+ T lymphocytes [7]. Neurological involvement has been described, including the detection of HHV-7 in central nervous system samples and reports of encephalitis, seizures, and other neurological syndromes [7,8,9,10].

Beyond acute infection, there is historical precedent for implicating herpesviruses in chronic post-viral syndromes. In myalgic encephalomyelitis/chronic fatigue syndrome (ME/CFS), active HHV-6 and HHV-7 infections have been reported in subsets of patients and have been associated with clinical outcomes and immune activation [11,12]. Antiviral treatment studies targeting herpesviruses have reported partial clinical benefit in selected patients, supporting continued investigation of viral reactivation as a potential contributor to disease pathophysiology [11,12,13,14].

Despite these observations, HHV-7 has rarely been systematically investigated in the context of long COVID. Systematic evidence on herpesvirus reactivation in COVID-19 has predominantly concerned EBV, CMV, HHV-6, HSV, and VZV, with HHV-7 largely absent from pooled analyses [5], leaving a potential gap in the current understanding of post-viral syndromes.

The present study was prompted by the observation of one patient who repeatedly tested positive for HHV-7 by polymerase chain reaction (PCR) following SARS-CoV-2 infection, with viral load fluctuations paralleling self-reported neurological and neuropsychiatric symptoms. This prompted a retrospective analysis comparing HHV-7 positivity in a historical pre-COVID-19 laboratory reference cohort with a cohort of patients presenting with a neurological/neuropsychiatric long COVID phenotype within 24 months of infection. The aim was to determine whether HHV-7 positivity appeared enriched in this clinical phenotype and to generate hypotheses for prospective investigation. In this patient-initiated study, patients independently sought diagnostic HHV-7 testing after learning about possible herpesvirus reactivation through scientific publications and discussions within long COVID patient communities.

## 2. Materials and Methods

### 2.1. Study Design and Cohorts

This patient-initiated study consisted of a retrospective observational analysis of pre-existing diagnostic laboratory records. The scientific hypothesis arose from observations and discussions within long COVID patient communities. No participant was prospectively enrolled under a research protocol, and no study-specific questionnaire, interview, intervention, or additional biological sampling was performed. Two cohorts were defined retrospectively from completed diagnostic records. The two cohorts comprised different individuals; no individual contributed data to both cohorts.

- Cohort A—Pre-COVID Historical Reference (2012–2019): A total of 320 archival diagnostic HHV-7 whole-blood PCR results from samples submitted for diagnostic purposes before the emergence of SARS-CoV-2. All eligible HHV-7 whole-blood PCR results available in the laboratory database from 2012 through 2019 were included; no symptom-based sampling or additional screening was applied. Testing was independent of the present study. This cohort was used as a historical laboratory reference rather than as a matched control group.
- Cohort B—Neurological/Neuropsychiatric Long COVID (≤24 months post-infection): A total of 30 patients (18 women and 12 men; age range, 21–55 years) with a reported history of SARS-CoV-2 infection documented by prior PCR or antibody testing and persistent neurological and/or neuropsychiatric symptoms following infection. Patients independently contacted the laboratory and voluntarily requested HHV-7 PCR testing to obtain an individual diagnostic result, following scientific publications and discussions within long COVID patient communities concerning possible HHV-6 and HHV-7 reactivation. Testing was not assigned or required by a research protocol, and each patient received an individual laboratory report. Only after diagnostic testing had been completed and the individual reports had been issued were the available results retrospectively reviewed and assembled into the present cohort.

For the purposes of this retrospective study, the term neurological refers to self-reported cognitive dysfunction, memory impairment, autonomic dysfunction, sensory hypersensitivity, and related neurological symptoms. The term neuropsychiatric refers to new-onset anxiety or panic symptoms, affective instability, insomnia, and internal agitation occurring after SARS-CoV-2 infection. These categories are descriptive and do not imply independently adjudicated neurological or psychiatric diagnoses. Cohort B was not selected using a prospectively administered standardized long COVID case-definition instrument, such as the World Health Organization (WHO) clinical case definition [15], and no standardized symptom questionnaire was administered. Accordingly, Cohort B should be regarded as a study-specific neurological/neuropsychiatric long COVID phenotype based on a retrospective patient-reported history. The 24-month window was selected to capture an early-to-intermediate post-infection period while remaining consistent with longitudinal studies characterizing symptom and recovery trajectories through 24 months after SARS-CoV-2 infection [16].

### 2.2. Clinical Material

Cohort A comprised 320 archival diagnostic laboratory tests from the pre-COVID-19 period (2012–2019). Cohort B comprised 30 peripheral whole-blood samples from 30 different patients (18 women and 12 men; age range, 21–55 years), collected in ethylenediaminetetraacetic acid (EDTA) tubes from patients reporting prior SARS-CoV-2 infection within the preceding 24 months. The cohorts were mutually exclusive, with no individual represented in both Cohort A and Cohort B.

All assays were performed on EDTA-treated whole blood as part of routine diagnostic workflows. Results were extracted retrospectively from the laboratory database after completion of diagnostic testing.

### 2.3. DNA Extraction and Quantitative PCR Conditions

Genomic DNA was extracted from whole blood using the phenol-chloroform method as described by Shan et al. [17]. TaqMan real-time quantitative PCR was performed as described by Fremont et al. [18]. The assay was performed on the FAST7500 instrument (Thermo Fisher Scientific, Merelbeke, Belgium); thermal cycling started with a 10 min incubation at 95 °C, followed by 45 cycles of 95 °C for 15 s and 60 °C for 1 min. Primers and probes were synthesized by Eurogentec (Seraing, Belgium). HHV-7, HHV-6, and EBV were tested in separate PCRs. A PCR inhibition control was included; no separate DNA extraction control was used.

### 2.4. Viral Load Determination

As described by Fremont et al. [18], viral load determination was based on relative quantification of the target sequence (viral DNA) and a reference sequence present in human genomic DNA, namely, a non-polymorphic region of the breast cancer 1 (BRCA1) locus present in two copies in a diploid genome. By comparing the cycle threshold (Ct) of the viral PCR with the cycle threshold of the reference PCR, the ratio of the number of viral copies to the number of cellular genomes (Ncop/Ncg) was calculated according to the formula Ncop/Ncg = [2^(Ct[Ref PCR] ∗ Ct[Viral PCR] + 1)]. Quantification for the present analysis therefore used relative Ct normalization to BRCA1 as described in the cited method.

For convenience, viral loads were expressed as the number of viral copies per million cells: Ncop/Mc = 10^6^ × Ncop/Ncg [18].

Results were expressed as the number of viral copies per million peripheral blood cells. According to the laboratory’s reference ranges, values between >0 and 50 copies per million cells could be detectable but were classified as negative according to the laboratory-defined positivity threshold; values above 50 copies per million cells were classified as positive. A similar qualitative interpretation (positive/negative) was applied for HHV-6 and EBV based on their respective assay thresholds.

EDTA whole blood was chosen over plasma for HHV-7 PCR because HHV-7 is largely cell-associated in CD4+ T cells and other leukocytes. Quantitative studies have demonstrated HHV-7 DNA in whole blood and peripheral blood mononuclear cells (PBMCs) [19]. In severe COVID-19 patients, Vilmane et al. reported HHV-7 DNA in 20.9% of whole-blood DNA samples versus 1.2% of plasma samples at baseline and 16.7% versus 6.25% on serial testing, highlighting the lower sensitivity of plasma for HHV-7 DNA detection [6].

### 2.5. Data Analysis

We summarized HHV-7 PCR positivity in the two cohorts using proportions and 95% confidence intervals (Wilson method). The primary comparison was the proportion of positive HHV-7 results in the neurological/neuropsychiatric long COVID cohort versus the historical pre-COVID-19 laboratory reference cohort. A two-sided Fisher’s exact test was used for the between-cohort comparison. Because Cohort A was not designed as a matched control group, the comparison was interpreted as exploratory and hypothesis-generating. Viral load distributions and self-reported symptom patterns within Cohort B were also summarized descriptively. Separately, archived routine laboratory positivity percentages for HHV-7, HHV-6, and EBV during 2012–2019 and from 2020 onward were summarized descriptively to provide temporal laboratory context. The post-2020 routine laboratory population was not treated as an additional clinical cohort and was excluded from inferential testing.

## 3. Results

### 3.1. HHV-7 Positivity in the Two Study Cohorts

**Figure 1:**
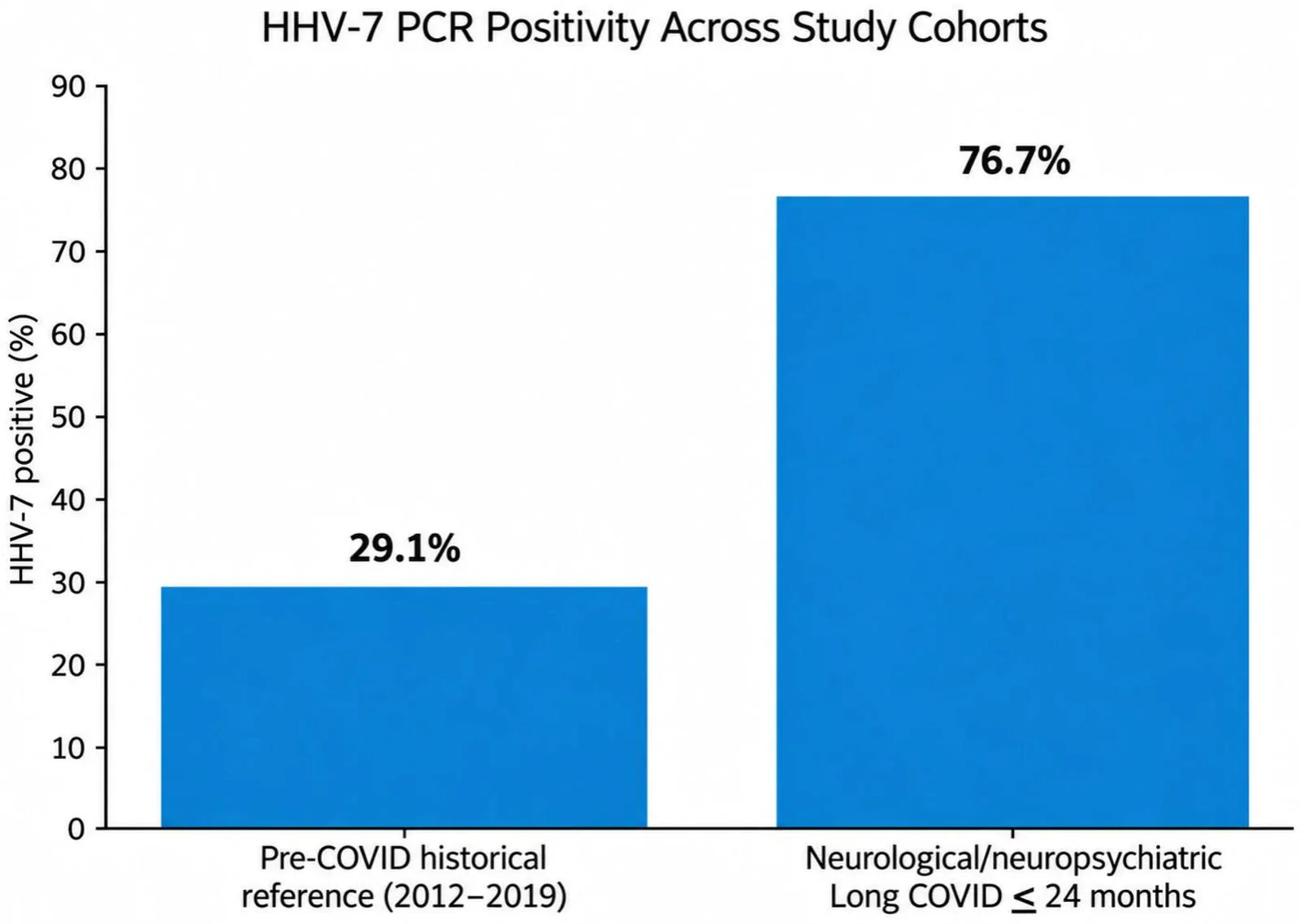
HHV-7 PCR positivity in the historical pre-COVID-19 reference cohort and the neurological/neuropsychiatric long COVID cohort.

HHV-7 positivity was 29.1% (93/320; 95% CI, 24.4–34.3%) in the historical pre-COVID-19 laboratory reference cohort and 76.7% (23 of 30 patients; 95% CI, 59.1–88.2%) in the neurological/neuropsychiatric long COVID cohort tested within 24 months of SARS-CoV-2 infection (Figure 1). The between-cohort difference was significant using a two-sided Fisher’s exact test (*p* < 0.000001). All six patients tested within the first 12 months after infection were HHV-7-positive (100%). Because the historical reference cohort was not matched to Cohort B, these findings should be interpreted as an exploratory association rather than a causal estimate.

### 3.2. Temporal Patterns of Other Herpesviruses in the Laboratory Database

To provide descriptive temporal laboratory context without defining an additional clinical cohort, archived routine diagnostic results from the same laboratory were reviewed for the pre-COVID-19 period (2012–2019) and for the period from 2020 onward. The post-2020 samples were submitted by individuals seeking diagnostic evaluation for non-specific complaints, including fatigue, and individual SARS-CoV-2 infection status was not systematically available. In these routine laboratory populations, HHV-7 positivity was 29.1% before 2020 and 54% from 2020 onward; HHV-6 positivity was 3% and 4%, respectively; and EBV positivity was 2% and 22.5%, respectively. These values are presented as descriptive temporal laboratory observations only. The post-2020 data were not treated as a clinical comparison cohort and were not included in the inferential analysis.

### 3.3. HHV-7 Viral Loads Among Positive Cases

All HHV-7-positive patients exhibited viral DNA levels well above the laboratory’s positivity threshold of 50 copies per million cells. The lowest quantified HHV-7 load was 96 copies/million cells, and the highest reached 2025 copies/million cells. Most patients had values above 250 copies/million cells. No borderline or low-positive cases were observed, suggesting that reactivation, when present, was virologically robust and not due to background noise or technical artifacts. This supports the clinical relevance of HHV-7 detection in these patients.

### 3.4. Neuropsychiatric Symptom Patterns and HHV-7 Status

A notable clinical pattern emerged: long COVID patients who tested positive for HHV-7 frequently reported significant psychiatric symptoms, including episodes of intense anxiety, panic attacks, and a chronic sense of internal agitation. Among HHV-7-positive long COVID patients, 21 of 23 (91.3%) reported such symptoms. Among the HHV-7-negative patients within this cohort, prominent anxiety or panic symptoms were not a consistent feature. Although causality cannot be established, this association raises the possibility that active HHV-7 replication may contribute to neuropsychiatric features in long COVID.

### 3.5. Index Case: Retrospective Review of Serial Viral Load and Symptoms

To illustrate the temporal relationship between HHV-7 activity and clinical evolution, we retrospectively reviewed serial laboratory results from one index patient (Table 1). Clinical symptoms, treatment history, and functional status were self-reported by the patient and were not independently assessed by the study team:

The index patient contracted SARS-CoV-2 infection in July 2023. The neurological/neuropsychiatric long COVID phenotype began between September and late October 2023 and included severe insomnia, nightmares, intense anxiety with panic episodes, rapid weight loss (from 55 to 50 kg), tachycardia, postural orthostatic tachycardia syndrome (POTS), sound hypersensitivity, memory impairment, and cognitive dysfunction.

Symptoms progressively worsened over the following months. By November 2024, the patient had lost the ability to work, leave her home, or maintain social interactions. The condition further deteriorated, reaching a peak in March 2025, at which point psychiatric hospitalization was considered due to the severity of neuropsychiatric symptoms.

Multiple oral treatments were attempted, including benzodiazepines, herbal anxiolytics (hawthorn, passionflower), ivabradine, low-dose naltrexone, and antihistamines. All resulted in paradoxical reactions, with marked exacerbation of symptoms lasting several days after each exposure.

In September 2025, the patient initiated antiviral therapy consisting of oral valganciclovir and artesunate (100 mg daily). Both agents have been evaluated as antiviral treatments for reactivated HHV-6/HHV-7 in ME/CFS [12]. Treatment continued for approximately two months but was discontinued due to poor tolerability and paradoxical reactions.

Subsequent testing in November 2025 showed a reduction in the HHV-7 viral load, with continued improvement through January 2026, including normalization of PCR results (Table 1). This virological response was accompanied by significant clinical improvement, including better sleep, reduced heart rate, improved heart rate variability (HRV), enhanced cognitive function, and partial restoration of social functioning, although physical weakness persisted.

In summary, this patient, infected with SARS-CoV-2 in July 2023, developed a progressive neurological/neuropsychiatric long COVID phenotype marked by brain fog, internal tremors, insomnia, and panic episodes. Serial HHV-7 PCR testing showed an undetectable viral load before symptom onset, followed by progressive viremia in parallel with self-reported symptom severity. Antiviral treatment administered from September to October 2025 was followed by partial clinical improvement and a reduction in viral load, although therapy was later discontinued due to side effects. Throughout the illness, EBV and HHV-6 PCRs remained negative.

This case illustrates a temporal association between the HHV-7 viral load and self-reported symptom expression in long COVID, echoing prior observations in ME/CFS in which suppression of herpesvirus activity was associated with clinical benefit [12,13,14].

## 4. Discussion

This retrospective study identifies a marked difference in HHV-7 positivity between a historical pre-COVID-19 laboratory reference cohort (29.1%, 93/320) and a cohort of patients with a neurological/neuropsychiatric long COVID phenotype tested within 24 months of SARS-CoV-2 infection (76.7%, 23/30). The between-cohort difference was significant using a two-sided Fisher’s exact test (*p* < 0.000001). The historical cohort was not matched to the long COVID cohort, so the comparison should be regarded as exploratory. Nevertheless, the magnitude of the difference supports prospective investigation of HHV-7 in this clinical phenotype.

Routine laboratory data provided a secondary temporal context for the primary comparison. Among samples submitted for non-specific complaints, including fatigue, HHV-7 positivity was 29.1% in 2012–2019 and 54% from 2020 onward; HHV-6 positivity was 3% and 4%, respectively; and EBV positivity was 2% and 22.5%, respectively. Thus, HHV-6 remained essentially stable across the two periods, while the post-2020 routine laboratory data showed higher HHV-7 and EBV positivity. Because individual SARS-CoV-2 infection status and detailed clinical phenotyping were not systematically available in this routine post-2020 population, these descriptive temporal differences cannot be attributed directly to SARS-CoV-2 infection. Importantly, HHV-7 positivity was still higher, at 76.7%, in the specifically defined neurological/neuropsychiatric long COVID cohort.

These findings extend prior observations of herpesvirus reactivation during acute COVID-19. Studies in hospitalized and critically ill patients have reported the reactivation of multiple herpesviruses, including HHV-7, often in association with disease severity and immune dysregulation [4,5,6]. Those studies largely described viral reactivation during the acute phase, whereas the present observations concern patients with persistent neurological and neuropsychiatric symptoms after the acute infection.

A potential biological contribution of HHV-7 is plausible given its CD4+ T-cell tropism and the neurological involvement described in the literature [7]. HHV-7 has been associated with serious neurological manifestations, including encephalitis, seizures, and other central nervous system syndromes [8,9,10]. These properties provide a biological rationale for investigating whether HHV-7 activity contributes to symptom persistence in a subset of patients after SARS-CoV-2 infection.

The clinical pattern observed within Cohort B further supports this hypothesis-generating signal. HHV-7-positive patients frequently reported neuropsychiatric symptoms, particularly anxiety, internal agitation, and sleep disturbance. These findings can be considered in the context of the broader literature describing neurological involvement during HHV-7 infection [7,8,9,10]. However, symptoms were self-reported, no standardized psychiatric instrument was administered, and causality cannot be inferred from this retrospective observational design.

The longitudinal index case provides an additional within-patient observation. Rising HHV-7 viral load paralleled worsening of self-reported symptoms, followed by a reduction in viral load and partial clinical improvement after antiviral treatment. This temporal pattern resembles observations in ME/CFS studies in which herpesvirus-directed antiviral therapy was associated with clinical benefit in selected patients [12,13,14]. The index case was not clinically assessed by the study team, and therefore cannot establish a causal treatment effect.

This study has several limitations. Its retrospective design and modest long COVID sample size limit generalizability, and referral and self-selection bias may have influenced absolute positivity rates. The historical pre-COVID-19 cohort was used as a laboratory reference and was not matched to Cohort B for age, sex, or clinical characteristics. Long COVID status and neurological/neuropsychiatric manifestations in Cohort B were based on patient-reported history rather than a prospectively administered WHO case-definition instrument, standardized symptom questionnaire, or independent clinical adjudication. No standardized neurological examination, psychiatric diagnostic instrument, SARS-CoV-2 persistence assay, neuroimaging, cerebrospinal fluid analysis, or immune profiling was performed as part of this study. These limitations preclude causal inference and prevent direct assessment of central nervous system HHV-7 infection. Prospective studies with predefined case criteria, matched controls, standardized clinical phenotyping, and longitudinal virological measurements will be required to validate the present findings.

## 5. Conclusions

In conclusion, HHV-7 positivity was substantially more frequent in our neurological/neuropsychiatric long COVID samples than in the historical pre-COVID-19 laboratory reference cohort. Together with the known neurotropic properties of HHV-7 and the symptom pattern observed in the long COVID cohort, this finding identifies HHV-7 as a candidate for further investigation in post-COVID-19 neurological and neuropsychiatric illness.

The present data are exploratory and do not establish causality. They support prospective studies examining whether HHV-7 reactivation is reproducibly enriched in well-phenotyped long COVID cohorts and whether changes in viral activity track clinical evolution.

Prospective studies incorporating matched controls, standardized long COVID definitions, longitudinal virological monitoring, neurological and neuropsychiatric phenotyping, and immune profiling will be essential to determine whether HHV-7 has value as a biomarker or therapeutic target in long COVID.

## Figures and Tables

**Table 1:** Longitudinal HHV-7 viral load and self-reported clinical status in the index case.

Clinical Status	HHV-7 Load (Copies/Million Cells)	Date
Pre-COVID-19/pre-symptom baseline	0	Jul 2022
Self-reported symptom onset	Not tested	Sep–Oct 2023
First documented HHV-7-positive PCR; clinical worsening	140	Nov 2024
Clinical worsening	205	Jan 2025
Peak severity	329	Mar 2025
Improvement post-treatment	96	Nov 2025
Significant clinical improvement	0	Jan 2026

## Data Availability

Anonymized data can be accessed at R.E.D. Laboratories SA, Belgium.

## Abbreviations

The following abbreviations are used in this manuscript:

Breast cancer gene 1	BRCA1
Cycle threshold	Ct
Epstein–Barr virus	EBV
Ethylenediaminetetraacetic acid	EDTA
Human herpesvirus 6	HHV-6
Human herpesvirus 7	HHV-7
Heart rate variability	HRV
Myalgic encephalomyelitis/chronic fatigue syndrome	ME/CFS
Peripheral blood mononuclear cells	PBMCs
Polymerase chain reaction	PCR
Postural orthostatic tachycardia syndrome	POTS
Severe acute respiratory syndrome coronavirus 2	SARS-CoV-2
Cytomegalovirus	CMV
World Health Organization	WHO
